# Detection of circular permutations by Protein Language Models

**DOI:** 10.1016/j.csbj.2024.12.029

**Published:** 2024-12-30

**Authors:** Yue Hu, Bin Huang, Chun Zi Zang, Jia Jie Xu

**Affiliations:** a School of Bioengineering, Qilu University of Technology (Shandong Academy of Sciences), Jinan, Shandong 250300, China; b School of Life Sciences, Yunnan Normal University, Kunming, Yunnan 650500, China; c Kyiv College, Qilu University of Technology (Shandong Academy of Sciences), Jinan, Shandong 250300, China

**Keywords:** Circular Permutation； Protein Language Models； Protein Structure Alignment

## Abstract

Protein circular permutations are crucial for understanding protein evolution and functionality. Traditional detection methods face challenges: sequence-based approaches struggle with detecting distant homologs, while structure-based approaches are limited by the need for structure generation and often treat proteins as rigid bodies. Protein Language Model-based alignment tools have shown advantages in utilizing sequence information to overcome the challenges of detecting distant homologs without requiring structural input. However, many current Protein Language Model-based alignment methods, which rely on sequence alignment algorithms like the Smith-Waterman algorithm, face significant difficulties when dealing with circular permutation (CP) due to their dependency on linear sequence order. This sequence order dependency makes them unsuitable for accurately detecting CP. Our approach, named plmCP, combines classical genetic principles with modern alignment techniques leveraging Protein Language Models to address these limitations. By integrating genetic knowledge, the plmCP method avoids the sequence order dependency, allowing for effective detection of circular permutations and contributing significantly to protein research and engineering by embracing structural flexibility.

## 1 Introduction

**Fig. 1:**
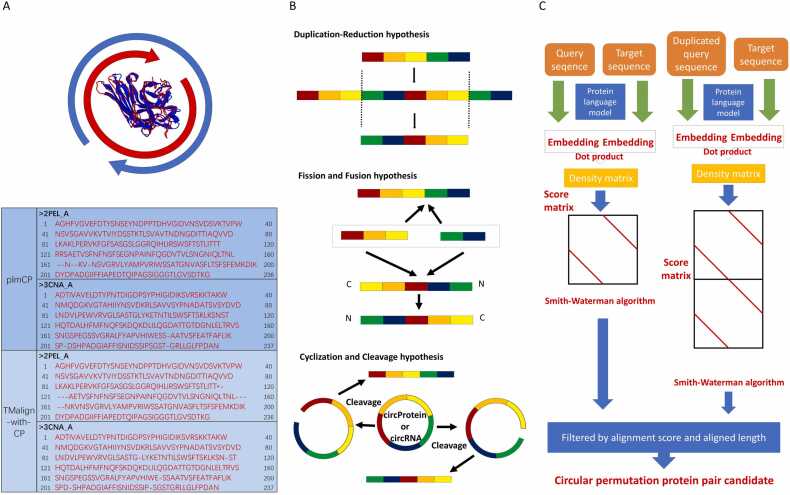
(A) Superposition of two circular permutation proteins (2PEL in blue and 3CNA in red) and the alignment results of TM-align and plmCP. (B) The possible mechanisms of circular permutation occur. Duplication-Reduction hypothesis: A gene is duplicated and redundant sequences are removed, creating a new sequence arrangement that preserves structure and function. Fission and Fusion hypothesis: A gene splits into two fragments that fuse in different configurations, potentially resulting in a sequence order distinct from the original while maintaining functionality. Cyclization and Cleavage hypothesis: Circular RNA or proteins are cleaved at various sites, allowing the fragments to reassemble into a circular permutation with normal structure and function. (C) The principal diagram of the plmCP.

Circular permutation in proteins [1], [2], [3], [4], [5], [6], [7], [8], [9] is a fascinating phenomenon that reflects the versatility and adaptability of these biological macromolecules. A circular permutation occurs when the order of amino acids in a protein's sequence is rearranged, such that the N-terminus and C-terminus are shifted (Fig. 1(A)). Despite this rearrangement, the overall three-dimensional shape and function of the protein often remain conserved (Fig. 1(A)). In proteins, they may occur through mechanisms such as gene duplication followed by the loss of redundant sections [1], or through fission and fusion events where partial proteins combine to form a new polypeptide [1]. And the third mechanism may occurred by the cyclization and cleavage of circRNA [10], [11], [12] or circProtein [13] (Fig. 1(B)). The duplication-reduction hypothesis (first picture in Fig. 1(B)) suggests that circular permutations (CP) occur when a gene undergoes duplication, followed by a breakage at specific points. Despite the existence of other potential mechanisms (other pictures in Fig. 1(B)), this hypothesis remains broad enough to explain all observed cases of circular permutation, making it a useful algorithm [4] (Fig. 1(C)) for detecting circular permutations (CP) formation. These permutations can lead to proteins with altered catalytic activities, increased thermosability, or novel functionalities [6], [7], [14]. The ability to detect and study such permutations enhances our understanding of protein evolution and enables the development of proteins with improved or novel characteristics.

Detecting circular permutations in proteins is challenging due to the non-linear nature of the rearrangement [1], [4], [15], [16], [17]. Algorithms for detecting circular permutations in proteins can be divided into sequence-based and structure-based methods. SeqCP [18] is a sequence-based algorithm for searching circularly permuted proteins. Its alignment followed by duplication-reduction hypothesis, which is the first occurrence mechanisms described above. It also has distinct advantages, one is fast calculation speed, and the other is that it does not need to know the structure of proteins. CE-CP [15], TM-align [19], [20] and CPSARST [21] are three typical structure-based methods for detecting circular permutations. Although CPSARST [21] is a structure-based method, in reality, it represents the information of the structure as a string, and when comparing the structure, it is similar to sequence alignment, which not only utilizes the accuracy of structure alignment but also the speed of sequence alignment.

However, the circular permutation detection method that relies solely on sequence dependencies, such as SeqCP [18], may be straightforward, but it struggles with accuracy, particularly for distantly related proteins with low sequence similarity. And the detection of such permutations relies on structural alignment methods, which require known three-dimensional structures and typically treat proteins as rigid bodies.

Protein Language Models [22], [23], [24], [25], [26] can help us extract structural information from the sequences in an implicit way. Protein Language Models [22], [23], [24], [25], [26] like ESM-1b [27], [28] and ESM2 [29], [30] are trained on large-scale sequence datasets (e.g., UniRef50 [31], [32]). They can capture structural information because they effectively learn the sequence context, sequence relationships, and long-range interactions, all of which are tightly linked to protein structure [25], [26]. By leveraging these deep sequence representations, Protein Language Models [22], [23], [24], [25], [26] (PLMs) provide insights that bridge the gap between sequence and structure. Protein alignment has been significantly enhanced by the use of Protein Language Models [22], [23], [24], [25], [26] (PLMs), with tools such as PLMAlign(an algorithm in PLMSearch) [33], DeepBlast [34], pLM-BLAST [35], and DEDAL [22]. A typical example is PLMAlign [33], which has shown promising results by using the inner product of amino acid vectors to construct a density matrix and a subsequent scoring matrix. This matrix is then utilized by the Smith-Waterman algorithm [36], [37], [38] to find the best local alignment between sequences. However, methods like PLMAlign [33] are inherently reliant on the linear order of sequences, making them less effective for detecting circular permutations. To address these limitations, we developed plmCP, a novel method that integrates a classical genetics-inspired circular permutation detection algorithm with PLMs. Similar to other methods utilizing Protein Language Models [22], [23], [24], [25], [26] for alignment, plmCP employs these models to generate embeddings, calculates inner products to construct a density matrix, and derives a scoring matrix for alignment using the Smith-Waterman algorithm [36], [37], [38]. However, to specifically detect circular permutations, plmCP followed the duplication-reduction hypothesis [1], [4] (Fig. 1(B)). As shown in Fig. 1(C), plmCP doubles the query sequence and aligns it with the target. If the doubled query yields a higher score or longer alignment compared to the original query, this indicates the presence of a circular permutation, distinguishing plmCP in its capability to identify such structural variations. Our approach implements a zero-shot inference method [39] using Protein Language Models [22], [23], [24], [25], [26], eliminating the need for training on specific datasets. The unsupervised nature of this approach, akin to existing models like PLMalign [33], allows it to directly discover novel insights without supervision. This enables the prediction of circular permutations without relying on task-specific training or fine-tuning. By overcoming the dependency on linear sequence order, plmCP is capable of effectively exploring and detecting circular permutations in proteins. This approach allows for a more comprehensive and flexible detection of circular permutations, expanding the capabilities of protein sequence analysis.

## 2 Method

The plmCP model is designed to identify circular permutations in protein sequences by leveraging the power of Protein Language Models [22], [23], [24], [25], [26] and the principles of pairwise sequence alignment algorithm. The method was inspired by PLMAlign [33] and CPSARST [21]. The plmCP (Fig. 1 (C)) employs the Protein Language Models [22], [23], [24], [25], [26] such as ESM-1b [27], [28] to generate per-residue embeddings. These embeddings represent the individual amino acids in the context of the entire protein sequence, capturing the intricate relationships and structural features that might not be evident from the primary sequence alone. The plmCP aligns the query sequence with the target sequence and vice versa. Additionally, it aligns a duplicate of the query sequence (or target sequence) with the target (or query). Using the inner product of amino acid vectors to construct a density matrix (Eq. (1)) and a subsequent scoring matrix (Eq. (2)). This matrix is then utilized by the Smith-Waterman algorithm [36], [37], [38] to find the best local alignment between sequences. Importantly, it changes to the classical backtracking algorithm in the Smith-Waterman algorithm [36], [37], [38] (Eq. (3)) compared with PLMAlign [33]. By comparing the results of these two alignments (the alignment score of the Smith-Waterman algorithm [36], [37], [38], the aligned length, and some parameters defined below), plmCP can detect circular permutations, which may arise from gene duplication followed by deletion events. The plmCP software is based on the codes of PLMAlign [33] and combined with the CPSARST [21] algorithm to detect circular permutations of proteins.

(1) $${{\mathrm{Density}=E}_{\mathrm{query}}}^{T}E_{\mathrm{target}}$$

(2) $$\mathrm{score}\left(i,j\right)=\max\left\{\begin{array}{c} \mathrm{score}(i-1,j)-gap\\ \mathrm{score}(i,j-1)-gap\\ \mathrm{score}\left(i-1,j-1\right)+\mathrm{density}(i,j)\\ 0 \end{array}\right)$$

(3) $$\mathrm{track}\mathrm{back}（i，j）=\left\{\begin{array}{c} 3,\mathrm{if score}\left(i,j\right)==\mathrm{score}(i-1,j)-gap\;\\ 2,\mathrm{if score}\left(i,j\right)==\mathrm{score}(i,j-1)-gap\\ 1,\mathrm{if score}\left(i,j\right)==\mathrm{score}\left(i-1,j-1\right)+\mathrm{density}(i,j)\\ 0,\mathrm{if score}\left(i,j\right)==0 \end{array}\right)$$

Our program supports three large language models: ESM-1b [27], [28], ESM2 [29], [30], and ProtT5 [40]. We utilize the ESM models, specifically ESM-1b [27], [28] and ESM2 [29], [30], which are both transformer-based architectures consisting of 33 layers and approximately 650 million parameters. Each of these models generates a 1280-dimensional embedding for every amino acid in the input sequence. For ESM-1b [27], [28], we use the model version ESM1b_t33_650M_UR50S.pt, while for ESM2 [29], [30], we utilize ESM2_t33_650M_UR50D.pt. To ensure accuracy, the embeddings are computed at FP32 precision. Additionally, we integrate ProtT5 [40] using the prot_t5_xl_uniref50 model, which generates 1024-dimensional embeddings for each amino acid, providing complementary and comparative analysis. Unless otherwise specified, the default model used in our analysis is ESM-1b [27], [28].

Protein Language Models [22], [23], [24], [25], [26], including ESM-1b [27], [28], face inherent limitations regarding the maximum token length they can process in a single pass. To overcome this limitation, we implemented a sliding window technique, inspired by trRosettaX-Single [41], [42], specifically for processing sequences that exceed the token limit. This method divides long sequences into overlapping segments, ensuring that essential information, particularly at the termini, is preserved across segments. Each segment is processed individually, and their outputs are later combined. By doing so, we maintain the model's ability to capture the full sequence while mitigating the impact of the token length restriction. To ensure consistency and accuracy across segments, a normalization step is applied. Since each position in the sequence is covered by multiple overlapping sliding windows, the contributions from each window are assigned an equal weight of 1 and normalized by the total number of overlapping windows. This approach allows for accurate and reliable results, even when processing sequences significantly longer than the token limit. In our software, the slide window model is automatically applied when the sequence length exceeds 1000. As shown in the Supplementary text (2. Slide Windows detail), we employed a double-window strategy to slide over different parts of the sequence. Due to the 1000-length limit, each window has a maximum length of 500 amino acids. We tested various window sizes (300, 400, 500) and different grid divisions (100, 200, 300) for ESM-1b [27], [28]. We extracted all protein pairs in the CPDB(a database of circular permutation in proteins) database [2] with lengths exceeding 500 amino acids for sliding window (slide windows) analysis, as sequence duplication (length doubling), a key step in the plmCP algorithm, results in sequences exceeding 1000 amino acids. The results showed that, for most window and grid parameter configurations, the assessment of circular permutation (CP) was consistent. However, in a few cases, when the window length and grid size were multiples of each other, the reduced overlap led to slight deviations in the results. Among all tested configurations, the combination of window length 500 (w500) and grid size 300 (g300) achieved alignment with most assessments while maintaining low computational demands (Supplementary text: 2. Slide Windows detail). As a representative case, we present the alignment results for 2mta_H [43] and 1kv9_A [44] (see Supplementary Text). It is important to note that circular permutation protein pairs with lengths greater than 500 are exceedingly rare in the CPDB database [2] (4169 pairs in total), with only 22 such pairs available for analysis. By default, the window length is set to 500, with a slide shift length of 300, ensuring comprehensive coverage of the sequence without excessive computational overhead.

**Fig. 2:**
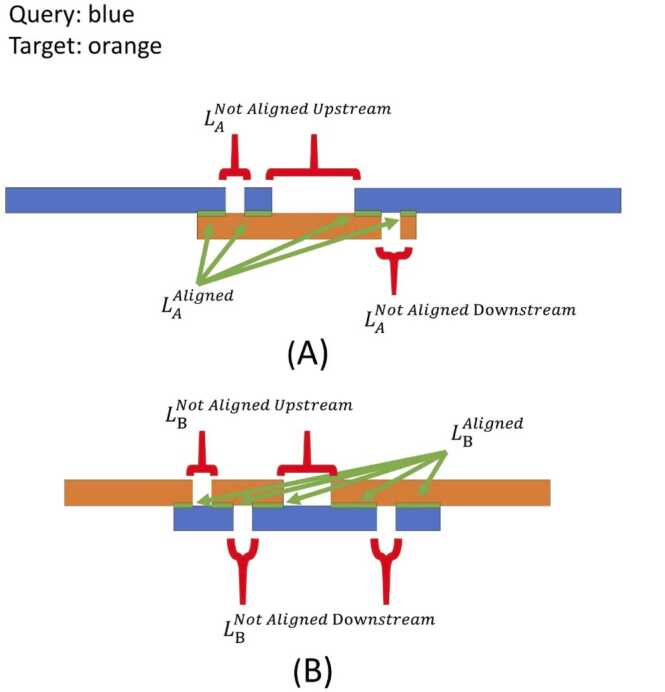
Schematic diagram of parameter definition: Query is represented in blue, and target is represented in orange. L_A is the aligned length obtained when the query sequence is doubled and aligned against the target, while L_B is the aligned length obtained when the target sequence is doubled and aligned against the query. L_A and L_B are the total alignment lengths, including gaps (marked by red brackets) and the aligned portions (indicated by green arrows pointing to green segments). The gaps are further categorized based on upstream and downstream contributions. The input sequences for query and target are defined and remain consistent, with both forward and reverse order taken into consideration in our program. The original input length of the query is L_query, and the original input length of the target is L_target, both of which remain unchanged.

To evaluate the quality of the identified circular permutations, we first defined some simple parameters aimed at performing a rough screening. It is important to note that due to the structural complexity and the frequent interdependence of circular permutations, there is no universal standard. Researchers should interpret results based on specific protein pairs and research context. We provide these parameters to assist researchers in making these determinations. As shown in Fig. 2, these parameters help in the initial identification and filtering of potential circular permutations, ensuring that further analysis can focus on more accurate and reliable results. In an ideal state, except for the end positions, two circular permutation proteins should be completely overlapping. In this case, the value of CP1 (Eq. (4)) should be equal to 1, and the value of CP2 (Eq. (5)) should be equal to 0. CP1 and CP2 reflect the overall alignment situation, and their sum theoretically equals 1. To simplify, we can select one of them as the overall metric. In the meantime, we should know if one protein aligns with itself, the value of CP1 will also be equal to 1, and the value of CP2 will also be equal to 0. Indeed, the protein may be circularly permutated from itself. In this circumstance, the alignment will be from the beginning. We can check the alignment to exclude this case. It is important to note that circular permutation often does not occur in isolation. it can accompany other structural variations such as insertions, deletions, and repetitions. These variations can lead to redundancies in the alignment, making certain regions misaligned. To accommodate these structural variations, we can assess each protein's alignment specifically. To analyse the alignment of each sequence individually, we defined 6 parameters ((6), (7), (8), (9), (10), (11)). (6), (9) reflect the aligned proportion compared to the target length or query length. (7), (10) reflect the insertion of upstream sequences compared to target length or query length. (8), (11) reflect the insertion of upstream sequences compared to target length or query length. For instance, $CP_{\mathrm{chec}k_{A}}$ reflects the alignment situation of protein A, while $CP_{\mathrm{chec}k_{\mathrm{AN}1}}$ and $CP_{\mathrm{chec}k_{\mathrm{AN}2}}$ indicate areas that did not align. Theoretically, the sum of these three values is approximately 1, and they are closely related. If CP_ $CP_{\mathrm{chec}k_{A}}$ is close to 1, it signifies a strong presence of circular permutation in protein A. Similarly, if CP_ $CP_{\mathrm{chec}k_{B}}$is not close to 1, it suggests that other structural variations have occurred in protein B. For simplicity, we can primarily focus on $CP_{\mathrm{chec}k_{A}}$ or $CP_{\mathrm{chec}k_{B}}$as the key metric.

(4) $$\mathrm{CP}1=\frac{L_{A}^{\mathrm{Aligned}}*L_{B}^{\mathrm{Aligned}}}{L_{\mathrm{target}}*L_{\mathrm{query}}}$$

(5) $$\mathrm{CP}2=\frac{{(L}_{\mathrm{target}}-L_{A}^{\mathrm{Aligned}})*(L_{\mathrm{query}}-L_{B}^{\mathrm{Aligned}})}{L_{\mathrm{target}}*L_{\mathrm{query}}}$$

(6) $$CP_{\mathrm{chec}k_{A}}=\frac{L_{A}^{\mathrm{Aligned}}}{L_{\mathrm{target}}}$$

(7) $$CP_{\mathrm{chec}k_{\mathrm{AN}1}}=\frac{L_{A}^{\mathrm{NotAlignedUpstream}}}{L_{\mathrm{target}}}$$

(8) $$CP_{\mathrm{chec}k_{\mathrm{AN}2}}=\frac{L_{A}^{\mathrm{NotAlignedDown}\mathrm{stream}}}{L_{\mathrm{target}}}$$

(9) $$CP_{\mathrm{chec}k_{B}}=\frac{L_{B}^{\mathrm{Aligned}}}{L_{\mathrm{query}}}$$

(10) $$CP_{\mathrm{chec}k_{\mathrm{BN}1}}=\frac{L_{B}^{\mathrm{NotAlignedUpstream}}}{L_{\mathrm{query}}}$$

(11) $$CP_{\mathrm{chec}k_{\mathrm{BN}2}}=\frac{L_{B}^{\mathrm{NotAlignedDownStream}}}{L_{\mathrm{query}}}$$

In the evaluation of the plmCP model, we utilized the Protein Data Bank [45] (PDB) entries 3CNA [46] (concanavalin) and 2PEL [47] (peanut lectin) as the test case to demonstrate the efficacy of our method. We also validated our method using the RIPC database [48], [49]. The RIPC database [48], [49] is a diverse and challenging dataset that includes a wide range of protein families. It covers four major types of structural variations [48], [50]: Repetitions, Insertions, Circular Permutations, and Conformational variability. This database also provides reference information for correct alignment [48], [49]. A "correctly aligned position" in the context of the RIPC database [48], [49] is defined as the accurate correspondence of homologous residues (both the position and type are completely consistent) between two protein structures, accounting for the different types of structural variations present in the dataset. Correct alignment is established by maximizing structural similarity, and sequence similarity while also taking into consideration functionally important regions, like catalytic or binding sites, where applicable. This diversity makes RIPC database [48], [49] a robust dataset for evaluating structural alignment methods, and it is widely used in the field, including by recent tools like ICARUS [49]. To further test our approach, we also utilized the CPDB (a database of circular permutation in proteins) dataset [2], which contains a total of 4169 pairs of circular permutation proteins. Since there were no reference alignment results available in this database, we primarily used the TM-align program [20] for comparison.

In our study, we chose a structure-based approach (i.e., TM-align [20]) to provide a clear comparison to sequence-based methods. Since TM-align [20] inherently has features that address circular permutation, it serves as a useful point of reference for evaluating our sequence-based method. TM-align [20] and TM-score [20] are currently considered the gold standard for structural alignments, while our method relies on sequence alignment. We believe this direct comparison helps to clearly illustrate the distinct applicability and strengths of each approach. We primarily used TM-align [20] for comparison, and in some specific cases, we also used software such as CE [51], CE-CP [15], FATCAT(rigid) [52], [53], [54], FATCAT(flexible) [52], [53], [54], SeqCP [18], CPSARST [21], and ICARUS [49]. CE [51], CE-CP [15], FATCAT(rigid) [52], [53], [54], and FATCAT(flexible) [52], [53], [54] were calculated using the online server at https://www.rcsb.org/alignment with default parameters. SeqCP [18] and CPSARST [21] were also calculated using the online server with default parameters. TM-align [20] and ICARUS [49] were calculated using locally installed software packages with default parameters.

We have included the detailed performance comparison of ESM-1b [27], [28], ESM2 [29], [30], and ProtT5 [40] in Supplementary Table 1, with specific statistical results provided in Supplementary Tables 2, 3, and 4, respectively. We have included the SlideWindow tests in the Original_results section of the supplement. The program ran perfectly and achieved the intended goals. We have also provided all source code in the plmCP_source_code section and all raw results in the Original_results section of the supplementary materials.

## 3 Results

First, we utilize two remote evolutional proteins 3CNA [46] and 2PEL [47] (sequence identity about 15 %) as the case to verify the ability to detect the remote circular permutation by plmCP. The TM-align [20] tool compares those two proteins using two methods: one with circular permutation (TM-score=0.91) and the other without (TM-score=0.48). These methods yield significantly different results. With circular permutation, the two proteins superpose perfectly, indicating that the TM-align [20] tool can accurately detect this circular permutation pair (Fig. 1(A)). Next, we employ plmCP to analyse the same proteins, obtaining the CP1 value (0.95) is approximately 1 and the CP2 value (0.00065) is close to 0 (see the definition of parameters in Method), suggesting that these proteins may indeed undergo circular permutation. Finally, when comparing the alignments from TM-align [20] (with CP parameter) and plmCP, we observe obvious similarities between the two methods. (Fig. 1(A)).

We then utilize another protein pairwise comparison tool (CE [51], CE-CP [15], FATCAT(rigid) [52], [53], [54], and FATCAT(flexible) [52], [53], [54]) to superpose the proteins and compute the TM-score, which gives a TM-score of 0.22, 0.32, 0.15, 0.09, respectively. CPSARST [21] can identify the relationship when the query is 2PEL [47], but it does not find any relation when searching with 3CNA [46]. The sequence-based method, seqCP, fails to detect any relationship between the proteins. These tools perform poorly with those protein pairs.

To further validate our approach, we employed a challenging database (the RIPC database [48]) that specifically contains protein structures with circular permutations. First, we focused on the performance of plmCP, TM-align [20], and ICARUS [49]. In the case of 1NLS [55], [56] and 2BQP [57], we demonstrated consistency among the three programs. In another case of 1NW5 [58] and 2ADM [59], our program plmCP performed exceptionally well, outperforming both TM-align [20] (which has a very low TM-score [20]) and ICARUS [49]. Those protein pairs have a context of circular permutation and insertion structural variations, which will make it hard to align. It's worth noting that ICARUS [49] incurs significant computational costs. Compared with reference of structures contained circular permutation type in the RIPC database [48], [49], the plmCP archived a mean percentage score of 65.32–75.36 % accurately aligned positions by different PLMs (ESM-1b [27], [28], ESM2 [29], [30] and ProtT5 [40]), while the structure methods range from a mean of 27.85–76.05 % (Supplementary Table 1–4). This proves the effectiveness of plmCP software in detecting circular permutations. Beyond analysing the plmCP's performance in circular permutation detection, we also compared other types of structural variations in the database to the reference results. Compared with reference, the plmCP achieved a mean percentage score of 71.99–82.17 % accurately aligned positions by different PLMs(ESM-1b [27], [28], ESM2 [29], [30] and ProtT5 [40]), while the structure methods range from a mean of 56.6–81.2 % (Table 1, Supplementary Table 1–4). Our evaluation demonstrates that the performance of ESM-1b [27], [28], ESM2 [29], [30], and ProtT5 [40] is largely comparable, with ESM-1b [27], [28] exhibiting marginally superior results.

**Table 1:** Alignment of plmCP compared to reference in the RIPC database.

**Protein 1**	**Protein 2**	**Structure types**	**plmCP**		
			**ESM-1b**	**ESM2**	**ProtT5**
d1hcy_2	d1lnlb1	I	50.00 %	50.00 %	50.00 %
d1ay9b_	d1b12a_	I	90.00 %	90.00 %	90.00 %
d1gbga__	d1ovwa_	I	100.00 %	0.00 %	66.67 %
d1crl__	d1ede__	I	66.67 %	66.67 %	66.67 %
d1jj7a_	d1lvga_	I C	100.00 %	100.00 %	100.00 %
d2adma_	d2hmyb_	I	100.00 %	100.00 %	100.00 %
d1an9a1	d1npx_1	I	54.55 %	18.18 %	54.55 %
d1nkl__	d1qdma1	P	100.00 %	100.00 %	100.00 %
d1nls__	d2bqpa_	P	100.00 %	83.33 %	83.33 %
d1qasa2	d1rsy__	P	85.33 %	94.67 %	97.33 %
d1b5ta_	d1k87a2	PR	0.00 %	0.00 %	0.00 %
d1jwyb_	d1puja_	PI	91.67 %	75.00 %	66.67 %
d1jwyb_	d1u0la2	PI	100.00 %	100.00 %	90.91 %
d1nw5a_	d2adma_	PI	100.00 %	46.15 %	100.00 %
d1gsa_1	d2hgsa1	PC	60.00 %	40.00 %	40.00 %
d1qq5a_	d3chy__	PI	33.33 %	33.33 %	0.00 %
d1kiaa_	d1nw5a_	PI	83.33 %	83.33 %	75.00 %
d2bbma_	d4cln__	C	100.00 %	100.00 %	99.32 %
d1dlia1	d1mv8a1	C	100.00 %	100.00 %	100.00 %
d1hava_	d1kxf__	IC	75.00 %	75.00 %	100.00 %
d1l5ba_	d1l5ea_	C	100.00 %	100.00 %	99.01 %
d1ggga_	d1wdna_	C	100.00 %	100.00 %	100.00 %
d1d5fa_	d1nd7a_	IC	100.00 %	100.00 %	100.00 %
**MEAN**			82.17 %	71.99 %	77.37 %

Structure types: R is Repetitions, I is Insertions, P is Circular Permutation, C is Conformational Variability. The numbers in Table 1 represent the percentage of correctly aligned positions.

We conducted a simple statistical analysis. We performed a statistical analysis of these results using ANOVA (Analysis of variance) (see Supplementary text). For Table 1, we analysed the overall performance of plmCP using different Protein Language Models [22], [23], [24], [25], [26]. We found that the results indicate that our plmCP, using three different models, shows no significant internal differences (P = 0.548). Specifically, using the circular permutation structures from the RIPC database [48], [49], we performed a focused statistical test on the performance of different programs in circular permutation, using ANOVA to test Supplementary Table 1(E). Statistically, there is also no significant difference in performance between plmCP (including all three models) and the better-performing ICARUS [49]. Using a p-value threshold of 0.05, our model shows significant differences compared to several moderately performing models (Such as FATCAT [52], [53], [54], see 1.3.3 in the Supplementary text, highlighted in red and bold).

The example of the 3CNA [46] and the 2PEL [47] were chosen because they have been extensively studied. And the RIPC database [48], [49] was included as it provides a standard reference position. To verify how performance scales with model size across different lengths, and to validate the plmCP on a larger dataset, we conducted a study on the CPDB database [2] using ESM2's plmCP. The lengths in this database range from 24 to 665, and our algorithm is capable of detecting these circular permutation phenomena. Our filtering approach involves checking whether the alignment score increases after doubling the query length, while also ensuring that the alignment length increases—criteria indicative of circular permutation. Out of a total of 4169 circular permutation pairs, 4047 pairs met this criterion using our method. In comparison, using TM-align [20] as the benchmark, only 3540 pairs met the criterion. Based on these results, we found that the sequence length of the two proteins does not have a significant impact on our model's performance, which ensures the robustness of our approach. The analysis of CP1 values from 4047 protein pairs in CPDB using probability density estimation revealed a maximum peak at 0.88 and a one-sided confidence interval cutoff of 0.35. Protein pairs with CP1 > 0.88 can be considered highly reliable CP cases, while those between 0.35 and 0.88 indicate a moderate likelihood requiring further validation. (Supplementary text: 3. Probability Density Analysis of CP1 Values in CPDB)

## 4 Conclude and discussion

The results demonstrate that our method can perform comparative detection for various structural variations, confirming its universality and expanding its applicability. Overall, we proposed a sequence-based, non-linear, flexible protein alignment tool. The term "flexible" refers to our method's reliance on structural information derived from Protein Language Models [22], [23], [24], [25], [26], rather than solely depending on three-dimensional coordinates. This flexibility allows the alignment process to accommodate various structural variations effectively.

Many existing PLMs-based and sequence-based structural alignment tools do not consider the influence of sequence order on alignment. In our study, we address a significant gap in current sequence-based structural alignment tools, which often struggle to align structures when the sequence order differs, particularly in cases of circular permutation. Compared to first predicting the structure and then performing circular permutation analysis, this tool directly detects circular permutations in an end-to-end manner.

While our software demonstrates significant capabilities in protein alignment, we acknowledge that there are still many areas for improvement. Here, we discuss some key limitations of our method. First, as noted in Supplementary Table 1–4, plmCP does not always outperform existing tools such as ICARUS [49] on the RIPC database [48], [49]. In certain instances, ICARUS [49] achieves better results, particularly in structural alignment tasks involving circular permutations. This discrepancy arises because ICARUS [49] utilizes structural input, providing a more detailed view of protein conformations. In contrast, plmCP relies solely on sequence information, which, while computationally less demanding and more scalable, may not capture certain structural details as effectively. Nonetheless, this design choice allows plmCP to be applicable in scenarios where 3D structural data is unavailable, offering a practical alternative for sequence-based evaluations.

Additionally, one limitation of our approach is the inference latency associated with using large pre-trained language models (PLMs) to obtain static embeddings. The computational resources required for models with a high number of parameters lead to increased inference times, particularly when processing long input sequences. While optimizations like model pruning or sequence truncation exist, they often compromise the quality and accuracy of the embeddings, limiting the scalability and efficiency of our method in practical applications, especially with large datasets or real-time requirements.

Lastly, the effectiveness of our approach may vary depending on the choice of the model, as different PLMs are trained on distinct datasets and employ varied learning methods, which may lead to differences in the accuracy of embeddings for specific tasks. Our model operates in an end-to-end manner, limiting our access to the precision of intermediate embeddings, which means evaluations are based on overall performance rather than specific metrics. Our tests indicate that the performance of the models—ESM-1b [27], [28], ESM2 [29], [30], and ProtT5 [40]—is quite similar, demonstrating a certain level of robustness. However, the issue of model selection remains important and could impact specific use cases

Although our software has certain limitations, it offers significant potential applications, particularly in fields such as protein engineering, biotechnology, synthetic biology, and therapeutic protein development [7], [60], [61], [62]. Our method effectively addresses the challenges of circular permutation and other structural variations by leveraging Protein Language Models [22], [23], [24], [25], [26](PLMs). This capability allows our approach to handle a range of sequence and structural modifications, making it a flexible and versatile tool that supports a wide variety of protein research and design applications. Looking ahead, we see several exciting possibilities at the intersection of circular permutation and circRNA research. One area of exploration is whether circular permutation in proteins could originate from circular RNAs (circRNAs), and whether this connection can be experimentally validated. Furthermore, we propose that the algorithms developed for detecting protein circular permutation could potentially be adapted for circRNA identification, offering a novel approach to studying these RNA structures. In addition, we raise the question of whether overcoming sequence order dependency, as demonstrated in our circular permutation approach, could improve the accuracy of multiple sequence alignment (MSA). Given that MSA serves as the foundation for many structure prediction algorithms, improvements in alignment could lead to more accurate model predictions and a deeper understanding of protein and RNA structures.

## Author contributions

Hu Yue and Huang Bin secured funding and designed the algorithm, who are also guarantors of this work and, as such, had full access to all of the data in the study and takes responsibility for the integrity of the data and the accuracy of the data analysis; Hu Yue, Huang Bin, Zang ChunZi and Xu JiaJie performed computation and analyzed the data. All authors agree to the revised manuscript, the research does not involve any ethical projects requiring approval, all data are publicly available to the academic community, and the supported projects are listed above.

## CRediT authorship contribution statement

**Bin Huang:** Validation, Supervision, Methodology, Investigation, Conceptualization. **Chunzi Zang:** Validation, Investigation, Data curation. **Yue Hu:** Validation, Supervision, Software, Methodology, Investigation, Conceptualization. **JiaJie Xu:** Validation, Investigation.

## Declaration of Competing Interest

All authors declare no competing interests.

## Data Availability

Our software can be installed on a standard computer with a GPU, as demonstrated using an NVIDIA A4500 GPU, where inference for sequences of 400–600 residues takes a few minutes. We've also deployed an online version on Code Ocean. Our source code is available at https://github.com/YueHuLab/plmCP/, and it is now divided into four branches: (1) The branch using the ESM-1b model is available at https://github.com/YueHuLab/plmCP/tree/main (2) The branch using the ESM2 Protein Language Modelcan be accessed at https://github.com/YueHuLab/plmCP/tree/esm2 (3) The branch utilizing the ProtT5 Protein Language Modelis hosted at https://github.com/YueHuLab/plmCP/tree/ProtT5 (4) The SlideWindow branch is available at https://github.com/YueHuLab/plmCP/tree/SlideWindow For users who prefer not to install the software locally, we also provide an online version of the tool, which has been reviewed and validated, and can be accessed via Code Ocean at https://codeocean.com/capsule/8856481/tree/v1 All raw data and processed data handled by the TM-Align program and plmCP are available in the directory of “CPDB (Circular Permutation Database) database” at the following GitHub link (https://github.com/YueHuLab/plmCP/tree/main/). The file total_final_alignment_compare.txt contains all alignment results, including those obtained using the plmCP program, TM-Align considering circular permutation, and TM-Align without considering circular permutation.
