# Supplementary material for: Detection of circular permutations by Protein Language Models
Source: Comput Struct Biotechnol J. 2024 Dec 30;27:214–20. doi: 10.1016/j.csbj.2024.12.029 (PMC11757225; doi:10.1016/j.csbj.2024.12.029)

**1. Statistical Testing**

**1.1 Steps for Using ANOVA to Test Percentage Data**

(1) Convert to Proportions: Divide each percentage by 100 to get proportions between 0 and 1.

(2) Apply Arcsine Transformation: Use an arcsine square root transformation to make the data more suitable for ANOVA. Use an arcsine square root transformation to make the data more suitable for ANOVA by stabilizing variances and improving normality of proportion data, ensuring that it meets the assumptions of normality and homogeneity of variances. This is a commonly used method to handle percentage data, especially in statistical analyses involving ANOVA.

(3) Check Assumptions: Ensure normality and homogeneity of variances using appropriate tests.

(4) Run ANOVA: Perform one-way ANOVA to check for differences among groups.

(5) Post-Hoc Analysis (LSD method): If significant, conduct post-hoc tests to find which groups differ.

(6) Interpret Results: Interpret findings and report in context.

**1.2 Statistical Testing for Table 1 in Manuscript**

| **1.2.1 Test of Homogeneity of Variances** | | | |
| --- | --- | --- | --- |
|  | | | |
| Levene Statistic | df1 | df2 | Sig. |
| .446 | 2 | 66 | .642 |

The p-value of 0.642 indicates homogeneity of variances, which supports proceeding with the standard ANOVA process.

| **1.2.2 ANOVA** | | | | | |
| --- | --- | --- | --- | --- | --- |
|  | | | | | |
|  | Sum of Squares | df | Mean Square | F | Sig. |
| Between Groups | .265 | 2 | .133 | .608 | .548 |
| Within Groups | 14.396 | 66 | .218 |  |  |
| Total | 14.661 | 68 |  |  |  |

The p-value of 0.548 indicates that there is no significant difference in the results obtained by plmCP using three different protein language models (ESM1b, ESM2 and ProtT5).

**1.3 Statistical Testing for Supplementary Table 1 (E)**

| **1.3.1 Test of Homogeneity of Variances** | | | |
| --- | --- | --- | --- |
|  | | | |
| Levene Statistic | df1 | df2 | Sig. |
| 1.521 | 11 | 108 | .134 |

The p-value of 0.134 indicates homogeneity of variances, which supports proceeding with the standard ANOVA process.

| **1.3.2 ANOVA** | | | | | |
| --- | --- | --- | --- | --- | --- |
|  | | | | | |
|  | Sum of Squares | df | Mean Square | F | Sig. |
| Between Groups | 6.091 | 11 | .554 | 2.463 | .009 |
| Within Groups | 24.277 | 108 | .225 |  |  |
| Total | 30.367 | 119 |  |  |  |

The p-value of 0.009 indicates significant differences between groups, but further analysis is needed to determine which specific groups differ.

| **1.3.3 Multiple Comparisons** | | | | | | | |
| --- | --- | --- | --- | --- | --- | --- | --- |
| Dependent Variable:v3 | | | | | | | |
|  | (I) VAR00002 | (J) VAR00002 | Mean Difference (I-J) | Std. Error | Sig. | 95% Confidence Interval | |
|  |  |  |  |  |  | Lower Bound | Upper Bound |
| LSD | 1.00 | 2.00 | -.151685505 | .212030077 | .476 | -.57196590 | .26859489 |
|  |  | 3.00 | -.181990374 | .212030077 | .393 | -.60227077 | .23829002 |
|  |  | 4.00 | -.067324480 | .212030077 | .751 | -.48760488 | .35295592 |
|  |  | 5.00 | .350605994 | .212030077 | .101 | -.06967440 | .77088639 |
|  |  | 6.00 | .495466452^*^ | .212030077 | .021 | .07518606 | .91574685 |
|  |  | 7.00 | .127519635 | .212030077 | .549 | -.29276076 | .54780003 |
|  |  | 8.00 | .292392880 | .212030077 | .171 | -.12788752 | .71267328 |
|  |  | 9.00 | .285866488 | .212030077 | .180 | -.13441391 | .70614688 |
|  |  | 10.00 | -.217090541 | .212030077 | .308 | -.63737094 | .20318985 |
|  |  | 11.00 | -.065463662 | .212030077 | .758 | -.48574406 | .35481673 |
|  |  | 12.00 | -.043066008 | .212030077 | .839 | -.46334640 | .37721439 |
|  | 2.00 | 1.00 | .151685505 | .212030077 | .476 | -.26859489 | .57196590 |
|  |  | 3.00 | -.030304868 | .212030077 | .887 | -.45058526 | .38997553 |
|  |  | 4.00 | .084361025 | .212030077 | .692 | -.33591937 | .50464142 |
|  |  | 5.00 | .502291500^*^ | .212030077 | .020 | .08201110 | .92257190 |
|  |  | 6.00 | .647151957^*^ | .212030077 | .003 | .22687156 | 1.06743235 |
|  |  | 7.00 | .279205140 | .212030077 | .191 | -.14107526 | .69948554 |
|  |  | 8.00 | .444078385^*^ | .212030077 | .039 | .02379799 | .86435878 |
|  |  | 9.00 | .437551994^*^ | .212030077 | .041 | .01727160 | .85783239 |
|  |  | 10.00 | -.065405035 | .212030077 | .758 | -.48568543 | .35487536 |
|  |  | 11.00 | .086221844 | .212030077 | .685 | -.33405855 | .50650224 |
|  |  | 12.00 | .108619497 | .212030077 | .609 | -.31166090 | .52889989 |
|  | 3.00 | 1.00 | .181990374 | .212030077 | .393 | -.23829002 | .60227077 |
|  |  | 2.00 | .030304868 | .212030077 | .887 | -.38997553 | .45058526 |
|  |  | 4.00 | .114665894 | .212030077 | .590 | -.30561450 | .53494629 |
|  |  | 5.00 | .532596368^*^ | .212030077 | .013 | .11231597 | .95287676 |
|  |  | 6.00 | .677456825^*^ | .212030077 | .002 | .25717643 | 1.09773722 |
|  |  | 7.00 | .309510008 | .212030077 | .147 | -.11077039 | .72979040 |
|  |  | 8.00 | .474383253^*^ | .212030077 | .027 | .05410286 | .89466365 |
|  |  | 9.00 | .467856862^*^ | .212030077 | .029 | .04757647 | .88813726 |
|  |  | 10.00 | -.035100167 | .212030077 | .869 | -.45538056 | .38518023 |
|  |  | 11.00 | .116526712 | .212030077 | .584 | -.30375368 | .53680711 |
|  |  | 12.00 | .138924365 | .212030077 | .514 | -.28135603 | .55920476 |
|  | 4.00 | 1.00 | .067324480 | .212030077 | .751 | -.35295592 | .48760488 |
|  |  | 2.00 | -.084361025 | .212030077 | .692 | -.50464142 | .33591937 |
|  |  | 3.00 | -.114665894 | .212030077 | .590 | -.53494629 | .30561450 |
|  |  | 5.00 | .417930474 | .212030077 | .051 | -.00234992 | .83821087 |
|  |  | 6.00 | .562790932^*^ | .212030077 | .009 | .14251054 | .98307133 |
|  |  | 7.00 | .194844115 | .212030077 | .360 | -.22543628 | .61512451 |
|  |  | 8.00 | .359717360 | .212030077 | .093 | -.06056304 | .77999776 |
|  |  | 9.00 | .353190968 | .212030077 | .099 | -.06708943 | .77347136 |
|  |  | 10.00 | -.149766061 | .212030077 | .481 | -.57004646 | .27051433 |
|  |  | 11.00 | .001860818 | .212030077 | .993 | -.41841958 | .42214121 |
|  |  | 12.00 | .024258472 | .212030077 | .909 | -.39602192 | .44453887 |
|  | 5.00 | 1.00 | -.350605994 | .212030077 | .101 | -.77088639 | .06967440 |
|  |  | 2.00 | -.502291500^*^ | .212030077 | .020 | -.92257190 | -.08201110 |
|  |  | 3.00 | -.532596368^*^ | .212030077 | .013 | -.95287676 | -.11231597 |
|  |  | 4.00 | -.417930474 | .212030077 | .051 | -.83821087 | .00234992 |
|  |  | 6.00 | .144860457 | .212030077 | .496 | -.27541994 | .56514085 |
|  |  | 7.00 | -.223086359 | .212030077 | .295 | -.64336676 | .19719404 |
|  |  | 8.00 | -.058213114 | .212030077 | .784 | -.47849351 | .36206728 |
|  |  | 9.00 | -.064739506 | .212030077 | .761 | -.48501990 | .35554089 |
|  |  | 10.00 | -.567696535^*^ | .212030077 | .009 | -.98797693 | -.14741614 |
|  |  | 11.00 | -.416069656 | .212030077 | .052 | -.83635005 | .00421074 |
|  |  | 12.00 | -.393672002 | .212030077 | .066 | -.81395240 | .02660839 |
|  | 6.00 | 1.00 | -.495466452^*^ | .212030077 | .021 | -.91574685 | -.07518606 |
|  |  | 2.00 | -.647151957^*^ | .212030077 | .003 | -1.06743235 | -.22687156 |
|  |  | 3.00 | -.677456825^*^ | .212030077 | .002 | -1.09773722 | -.25717643 |
|  |  | 4.00 | -.562790932^*^ | .212030077 | .009 | -.98307133 | -.14251054 |
|  |  | 5.00 | -.144860457 | .212030077 | .496 | -.56514085 | .27541994 |
|  |  | 7.00 | -.367946817 | .212030077 | .086 | -.78822721 | .05233358 |
|  |  | 8.00 | -.203073572 | .212030077 | .340 | -.62335397 | .21720682 |
|  |  | 9.00 | -.209599963 | .212030077 | .325 | -.62988036 | .21068043 |
|  |  | 10.00 | -.712556992^*^ | .212030077 | .001 | -1.13283739 | -.29227660 |
|  |  | 11.00 | -.560930113^*^ | .212030077 | .009 | -.98121051 | -.14064972 |
|  |  | 12.00 | -.538532460^*^ | .212030077 | .013 | -.95881286 | -.11825206 |
|  | 7.00 | 1.00 | -.127519635 | .212030077 | .549 | -.54780003 | .29276076 |
|  |  | 2.00 | -.279205140 | .212030077 | .191 | -.69948554 | .14107526 |
|  |  | 3.00 | -.309510008 | .212030077 | .147 | -.72979040 | .11077039 |
|  |  | 4.00 | -.194844115 | .212030077 | .360 | -.61512451 | .22543628 |
|  |  | 5.00 | .223086359 | .212030077 | .295 | -.19719404 | .64336676 |
|  |  | 6.00 | .367946817 | .212030077 | .086 | -.05233358 | .78822721 |
|  |  | 8.00 | .164873245 | .212030077 | .439 | -.25540715 | .58515364 |
|  |  | 9.00 | .158346853 | .212030077 | .457 | -.26193354 | .57862725 |
|  |  | 10.00 | -.344610176 | .212030077 | .107 | -.76489057 | .07567022 |
|  |  | 11.00 | -.192983296 | .212030077 | .365 | -.61326369 | .22729710 |
|  |  | 12.00 | -.170585643 | .212030077 | .423 | -.59086604 | .24969475 |
|  | 8.00 | 1.00 | -.292392880 | .212030077 | .171 | -.71267328 | .12788752 |
|  |  | 2.00 | -.444078385^*^ | .212030077 | .039 | -.86435878 | -.02379799 |
|  |  | 3.00 | -.474383253^*^ | .212030077 | .027 | -.89466365 | -.05410286 |
|  |  | 4.00 | -.359717360 | .212030077 | .093 | -.77999776 | .06056304 |
|  |  | 5.00 | .058213114 | .212030077 | .784 | -.36206728 | .47849351 |
|  |  | 6.00 | .203073572 | .212030077 | .340 | -.21720682 | .62335397 |
|  |  | 7.00 | -.164873245 | .212030077 | .439 | -.58515364 | .25540715 |
|  |  | 9.00 | -.006526392 | .212030077 | .976 | -.42680679 | .41375400 |
|  |  | 10.00 | -.509483421^*^ | .212030077 | .018 | -.92976382 | -.08920302 |
|  |  | 11.00 | -.357856541 | .212030077 | .094 | -.77813694 | .06242385 |
|  |  | 12.00 | -.335458888 | .212030077 | .117 | -.75573928 | .08482151 |
|  | 9.00 | 1.00 | -.285866488 | .212030077 | .180 | -.70614688 | .13441391 |
|  |  | 2.00 | -.437551994^*^ | .212030077 | .041 | -.85783239 | -.01727160 |
|  |  | 3.00 | -.467856862^*^ | .212030077 | .029 | -.88813726 | -.04757647 |
|  |  | 4.00 | -.353190968 | .212030077 | .099 | -.77347136 | .06708943 |
|  |  | 5.00 | .064739506 | .212030077 | .761 | -.35554089 | .48501990 |
|  |  | 6.00 | .209599963 | .212030077 | .325 | -.21068043 | .62988036 |
|  |  | 7.00 | -.158346853 | .212030077 | .457 | -.57862725 | .26193354 |
|  |  | 8.00 | .006526392 | .212030077 | .976 | -.41375400 | .42680679 |
|  |  | 10.00 | -.502957029^*^ | .212030077 | .019 | -.92323742 | -.08267663 |
|  |  | 11.00 | -.351330150 | .212030077 | .100 | -.77161055 | .06895025 |
|  |  | 12.00 | -.328932496 | .212030077 | .124 | -.74921289 | .09134790 |
|  | 10.00 | 1.00 | .217090541 | .212030077 | .308 | -.20318985 | .63737094 |
|  |  | 2.00 | .065405035 | .212030077 | .758 | -.35487536 | .48568543 |
|  |  | 3.00 | .035100167 | .212030077 | .869 | -.38518023 | .45538056 |
|  |  | 4.00 | .149766061 | .212030077 | .481 | -.27051433 | .57004646 |
|  |  | 5.00 | .567696535^*^ | .212030077 | **.009** | .14741614 | .98797693 |
|  |  | 6.00 | .712556992^*^ | .212030077 | **.001** | .29227660 | 1.13283739 |
|  |  | 7.00 | .344610176 | .212030077 | .107 | -.07567022 | .76489057 |
|  |  | 8.00 | .509483421^*^ | .212030077 | **.018** | .08920302 | .92976382 |
|  |  | 9.00 | .502957029^*^ | .212030077 | **.019** | .08267663 | .92323742 |
|  |  | 11.00 | .151626879 | .212030077 | .476 | -.26865352 | .57190727 |
|  |  | 12.00 | .174024533 | .212030077 | .414 | -.24625586 | .59430493 |
|  | 11.00 | 1.00 | .065463662 | .212030077 | .758 | -.35481673 | .48574406 |
|  |  | 2.00 | -.086221844 | .212030077 | .685 | -.50650224 | .33405855 |
|  |  | 3.00 | -.116526712 | .212030077 | .584 | -.53680711 | .30375368 |
|  |  | 4.00 | -.001860818 | .212030077 | .993 | -.42214121 | .41841958 |
|  |  | 5.00 | .416069656 | .212030077 | .052 | -.00421074 | .83635005 |
|  |  | 6.00 | .560930113^*^ | .212030077 | **.009** | .14064972 | .98121051 |
|  |  | 7.00 | .192983296 | .212030077 | .365 | -.22729710 | .61326369 |
|  |  | 8.00 | .357856541 | .212030077 | .094 | -.06242385 | .77813694 |
|  |  | 9.00 | .351330150 | .212030077 | .100 | -.06895025 | .77161055 |
|  |  | 10.00 | -.151626879 | .212030077 | .476 | -.57190727 | .26865352 |
|  |  | 12.00 | .022397653 | .212030077 | .916 | -.39788274 | .44267805 |
|  | 12.00 | 1.00 | .043066008 | .212030077 | .839 | -.37721439 | .46334640 |
|  |  | 2.00 | -.108619497 | .212030077 | .609 | -.52889989 | .31166090 |
|  |  | 3.00 | -.138924365 | .212030077 | .514 | -.55920476 | .28135603 |
|  |  | 4.00 | -.024258472 | .212030077 | .909 | -.44453887 | .39602192 |
|  |  | 5.00 | .393672002 | .212030077 | .066 | -.02660839 | .81395240 |
|  |  | 6.00 | .538532460^*^ | .212030077 | **.013** | .11825206 | .95881286 |
|  |  | 7.00 | .170585643 | .212030077 | .423 | -.24969475 | .59086604 |
|  |  | 8.00 | .335458888 | .212030077 | .117 | -.08482151 | .75573928 |
|  |  | 9.00 | .328932496 | .212030077 | .124 | -.09134790 | .74921289 |
|  |  | 10.00 | -.174024533 | .212030077 | .414 | -.59430493 | .24625586 |
|  |  | 11.00 | -.022397653 | .212030077 | .916 | -.44267805 | .39788274 |
| *. The mean difference is significant at the 0.05 level. | | | | | | | |

1-12 are classified according to different software. Information can be found in the table below.

| ICARUS Level 1 | 1 |
| --- | --- |
| ICARUS Level 2 | 2 |
| ICARUS Level 3 | 3 |
| ICARUS Level 4 | 4 |
| TM-align | 5 |
| FATCT | 6 |
| DEDAL | 7 |
| KPAX | 8 |
| PAUL | 9 |
| plmCP ESM1b | 10 |
| plmCP ESM2 | 11 |
| plmCP ProtT5 | 12 |

The results indicate that our plmCP, using three different models, shows no significant internal differences. Statistically, there is also no significant difference in performance between plmCP (including all three models) and the better-performing ICARUs. Using a p-value threshold of 0.05, our model shows significant differences compared to several moderately performing models (highlighted in red and bold).

**2. Slide Windows details**

We tested different window lengths (300, 400 and 500) and shift lengths (100, 200 and 300) and found that the alignment length and score were generally consistent, demonstrating that our method has robustness within a certain range**.** Go to this directory (https://github.com/YueHuLab/plmCP/tree/SlideWindow) to check the log files. This test was conducted using 2mta_H and 1kv9_A, and can be run with test_1201_slide.py. The length of 1kv9_A is 664, and after duplication, it reaches 1328, which will trigger the slide windows setting. This program uses a double-window strategy to slide over different parts of the sequence. By combining each pair of windows, it extracts features to capture long-range interactions within the sequence. Each position is accessed multiple times to enhance the robustness of feature representation. The final result is obtained by averaging the features from multiple windows to achieve a more stable representation. The double-window approach allows the model to capture interactions between different parts of the sequence, enhancing the representation by providing richer context information. Below is a diagram showing the overlapping of sequences during feature extraction using double windows.
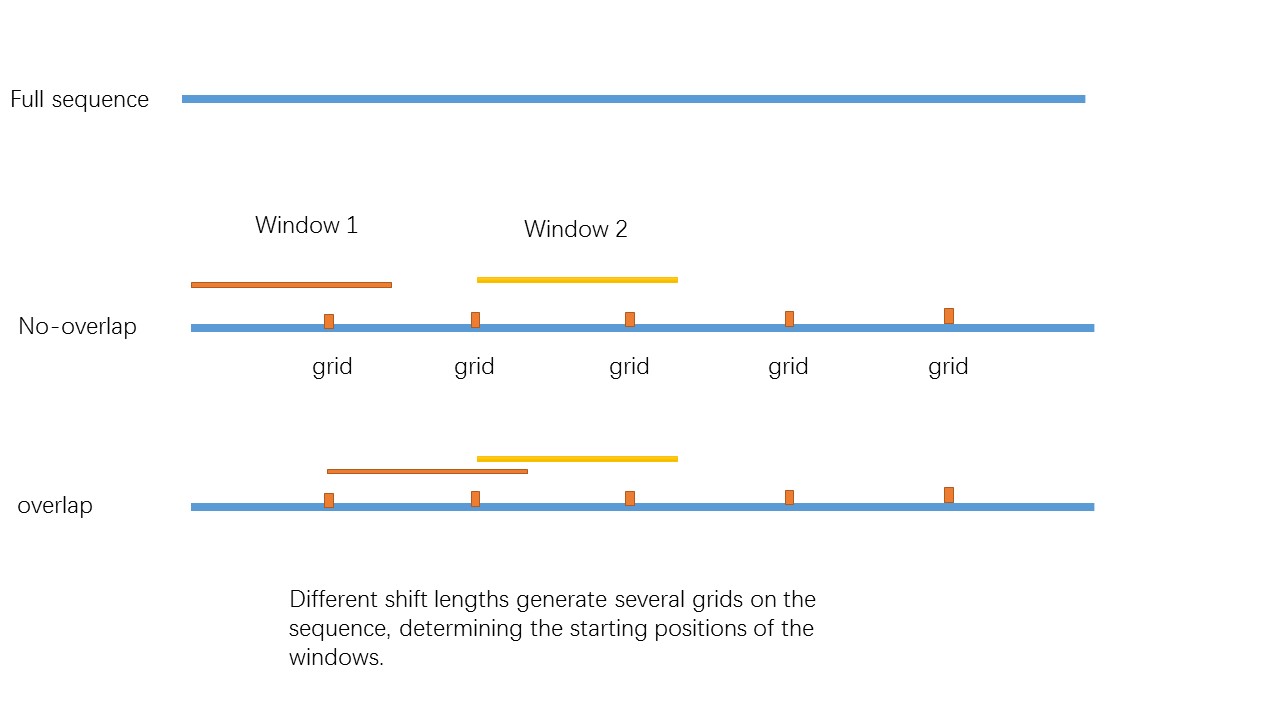


We tested various window sizes (300, 400, 500) and different grid divisions. For the test input library, sequences in CPDB longer than 500 amino acids underwent circular permutation (CP) analysis; duplication events can result in lengths exceeding 1000 amino acids. Such cases are rare, with only 22 protein pairs in CPDB exceeding the 500-amino acid threshold, all of which were tested. Protein pairs were evaluated using sliding windows of various lengths and grid divisions. In most cases, the method consistently identified circular permutations. However, when window lengths and grid sizes were multiples of each other, performance declined due to insufficient overlap. The configuration w500_g300 aligned with most assessments and required minimal computational resources.

3. Probability Density Analysis of CP1 Values in CPDB

The analysis of CP1 values from 4047 protein pairs in CPDB using probability density estimation revealed a maximum peak at 0.88 and a one-sided confidence interval cutoff of 0.35. Protein pairs with CP1 > 0.88 can be considered highly reliable CP cases, while those between 0.35 and 0.88 indicate a moderate likelihood requiring further validation.


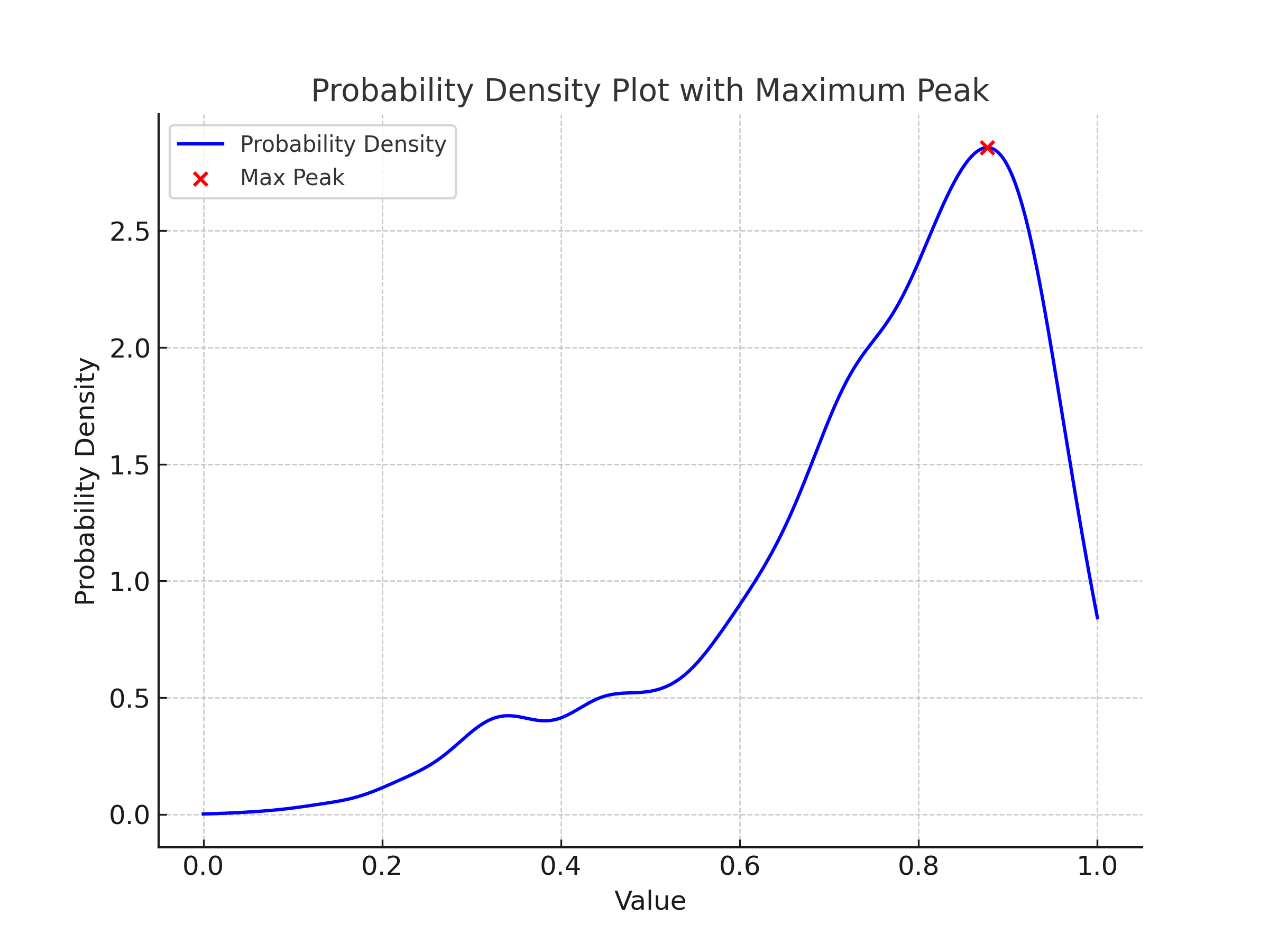

Supplement: Supplementary file 7 — Supplementary material [file mmc7.docx]
